# Signs of Selective Pressure on Genetic Variants Affecting Human Height

**DOI:** 10.1371/journal.pone.0027588

**Published:** 2011-11-09

**Authors:** Roberto Amato, Gennaro Miele, Antonella Monticelli, Sergio Cocozza

**Affiliations:** 1 Gruppo Interdipartimentale di Bioinformatica e Biologia Computazionale, Università di Napoli “Federico II” - Università di Salerno, Naples, Italy; 2 Dipartimento di Scienze Fisiche, Università degli Studi di Napoli “Federico II”, Naples, Italy; 3 Istituto Nazionale di Fisica Nucleare – Sezione di Napoli, Naples, Italy; 4 Istituto di Endocrinologia ed Oncologia Sperimentale, CNR Napoli, Naples, Italy; 5 Dipartimento di Biologia e Patologia Cellulare e Molecolare “L. Califano”, Università degli Studi di Napoli “Federico II”, Naples, Italy; Aarhus University, Denmark

## Abstract

Many decades of scientific investigation have proved the role of selective pressure in *Homo Sapiens* at least at the level of individual genes or loci. Nevertheless, there are examples of polygenic traits that are bound to be under selection, but studies devoted to apply population genetics methods to unveil such occurrence are still lacking. Stature provides a relevant example of well-studied polygenic trait for which is now available a genome-wide association study which has identified the genes involved in this trait, and which is known to be under selection. We studied the behavior of F_ST_ in a simulated toy model to detect population differentiation on a generic polygenic phenotype under selection. The simulations showed that the set of alleles involved in the trait has a higher mean F_ST_ value than those undergoing genetic drift only. In view of this we looked for an increase in the mean F_ST_ value of the 180 variants associated to human height. For this set of alleles we found F_ST_ to be significantly higher than the genomic background (p = 0.0356). On the basis of a statistical analysis we excluded that the increase was just due to the presence of outliers. We also proved as marginal the role played by local adaptation phenomena, even on different phenotypes in linkage disequilibrium with genetic variants involved in height. The increase of F_ST_ for the set of alleles involved in a polygenic trait seems to provide an example of symmetry breaking phenomenon concerning the population differentiation. The splitting in the allele frequencies would be driven by the initial conditions in the population dynamics which are stochastically modified by events like drift, bottlenecks, etc, and other stochastic events like the born of new mutations.

## Introduction

One of the greatest challenges in modern biology is to understand how selection has driven human evolution. Decades of work provided evidence of selective pressure in *Homo Sapiens* at the level of individual genes or loci [1]–[3]. Among the others, methods based on population differentiation were widely used to unveil their signature [4]. However, in most cases, variation in phenotype among individuals is the result of a polygenic effect, involving multiple genetic variations at multiple unlinked loci [5]. Up to now, only few studies investigated the presence of selective pressure on polygenic traits in humans. A possible explanation for this lack of evidence is that polygenic adaptation might be largely undetected by conventional methods able to look for selection [6]–[7]. Furthermore, the identification of signatures of polygenic evolution could require genome-scale data sources, with a well-defined set of genetic variants involved in the polygenic effect.

Stature is one of most studied polygenic traits, because measuring height is easy and replicable and its inheritance well recognized. Recently, genome-wide association studies contributed in the identification of genes involved in this trait. Lango Allen and colleagues, in particular, demonstrated that hundreds of genetic variants, in at least 180 loci, influence adult height [8]. Although this result explains approximately only 10% of the phenotypic variation in height, this study provides up to now the most detailed description of a polygenic effect in humans at molecular level.

From an evolutionary point of view, a complex interaction of different forces acts on stature and the complete dynamics is still not completely clear. In particular, several studies suggested a stabilizing selection on human height because of an increased number of health problems in very short and very tall individuals [9], [10]. At the same time, other studies invoked a directional sexual selection on male human height in that taller men often have more reproductive chances [11]. Anyway, a worldwide distributed sexual selection seems to represent an overall reasonable scenario, even though local adaptation phenomena that could favor particular heights and body shapes in particular environmental conditions cannot be excluded.

In this work we explored, in a simple simulated model, the behavior of F_ST_, a widely used method for detecting selection based on populations differentiation, on a generic polygenic trait. We then investigated whether a similar behavior was observable in a real case, namely in the set of loci related to height.

## Results and Discussion

To analyze the behavior of F_ST_ on a polygenic trait, we started by simulating the action of a polygenic stabilizing selection pressure in a very simple model. Such a model relies on the following simplifying assumptions: (i) each contributing allele has small and relatively equal additive effects, without neither environmental influences nor non-linear effects (dominance, epistasis, etc), (ii) individual fitness is given by a bell-shaped curve where the maximum is achieved by individual owning only a part of the advantageous alleles, (iii) no migration or demographic events affect the populations.

Under these simple assumptions, we observed that the set of alleles involved in the phenomenon has a higher mean F_ST_ value than those subject to genetic drift only. We thus checked whether the same behavior was observable in the 180 loci influencing adult height. Indeed, more than 80% of its variation within a given population is estimated to be attributable to additive genetic factors of small effects. Moreover, the authors found no evidence that non-additive effects including gene–gene interaction would increase the proportion of the phenotypic variance explained [8]. In the light of this, our simulations seem to reasonable model this scenario and, for this reason, we explicitly searched for an increase in the mean F_ST_ value of the 180 variants associated to height.

**Figure 1:**
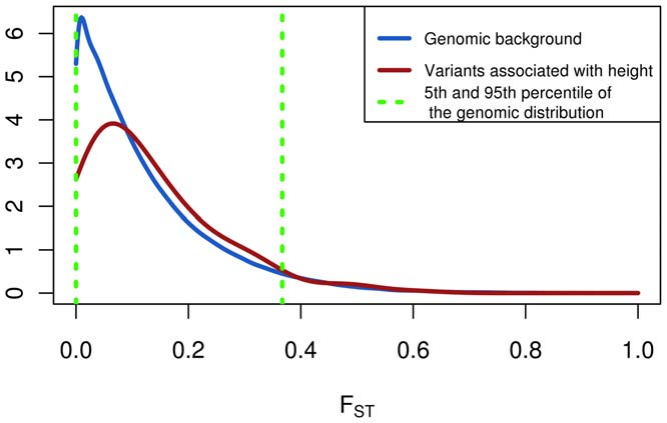
F_ST_ density distribution for the genomic background and the height associated variants. Green lines mark the 5^th^ and 95^th^ percentiles of the genomic distribution.

We found in the F_ST_ distribution for these variants an overrepresentation of higher values with respect to the genomic background (median = 0.1 vs. 0.086; p = 0.0356, one-tailed Mann-Whitney test; Figure 1). We investigated for potential confounding factors, first of all whether the increase was just due to the presence of outliers. We thus excluded from the initial set of height related variants those falling in the top or in the bottom 5% tail according to the genomic distribution of F_ST_, obtaining a set of 161 SNPs (hereafter denoted as “core set”). The number of outliers excluded is compatible with the expected 5% (4% and 5% of the SNPs falling in the top and in the bottom tail, respectively). Moreover, the “core set” still exhibits a significant overrepresentation of high values F_ST_ value (median = 0.1 vs. 0.086; p = 0.0232, one-tailed Mann-Whitney test).

In a recent paper, Pritchard and Di Rienzo argued that a signature of selection on a polygenic trait should reasonably be small and spread across the loci [12]. Our result is, to some extension, in agreement with this hypothesis. Indeed, the increased F_ST_ value is not dominated by few outlier loci, but small and distributed. From this point of view, also the low value of statistical significance that we found could be expected.

On the other hand, if we consider height either under a stabilizing selective pressure or a worldwide distributed sexual selection, the higher mean F_ST_ value could seem unexpected. Under these hypotheses, indeed, we expected a result that is opposite to that obtained, even in presence of marginal phenomena of local adaptation. In these conditions, one should expect the majority of the genes having a vanishing F_ST_ and some outliers with very high values of F_ST_. But looking at the simulated alleles trajectories over time, a possible explanation could be suggested. In simulations, one can observe that, in different populations, different sets of alleles become prevalent (Figure S1). In particular, the dynamics favors in each population the prevalence of a specific subset of alleles among the large amount of different subset choices all capable to maximize the phenotype under selective pressure. In absence of extraneous forces, the choice of the allele subset for each population is just randomly driven and, hence, it is highly probable to be different for each population. This results in an increase of the mean diversity among the populations.

This very simple model resembles the behavior of a well-known statistical mechanics phenomenon denoted as Spontaneous Symmetry Breaking (SSB). This mechanism generally plays a relevant role in system self-organization, and it is common in many fields of Natural Sciences where a system described in a theoretically symmetrical way ends up in a non-symmetrical state. The physics of condensed matter probably provides the most striking examples of SSB phenomena. In a ferromagnet cooled below its critical temperature, as the thermal fluctuations slow down, will become energetically favorable the appearance of domains where all elementary magnets point in the same direction, randomly chosen, and hence breaking the original rotational symmetry.

SSB has been widely observed in biological systems. Population genetics also provides examples of SSB even though in this case to lead the breaking are the initial conditions, stochastically modified by events like drift, bottlenecks, etc, and other stochastic events like the born of new mutations. Among the others, we can quote the role of symmetry breaking and coarsening in spatially distributed evolutionary processes relevant for genetic diversity and species formation [13]., and the relevance of symmetry breaking in the long-term evolution of multilocus traits [14].

**Figure 2:**
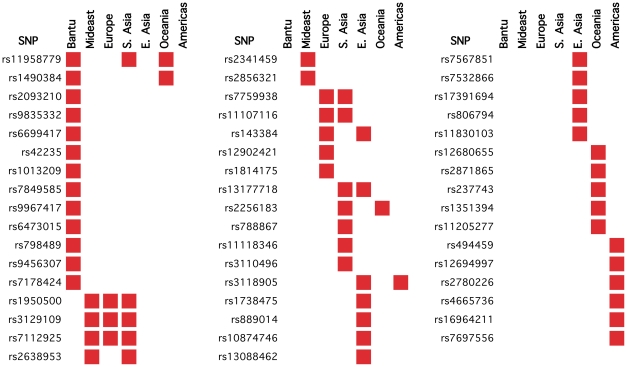
Variants associated to height with high iHS score. Red squares indicate mark the population in which the iHS score for the variant falls in the top 5^th^ percentile of the respective distribution.

The symmetry-breaking scenario could represent a simple yet reasonable model of the selection acting on height. But, even though height is basically under stabilizing selection, local adaptive phenomena cannot be excluded. To explicitly explore the presence of local adaptive phenomena, we analyzed how iHS, another marker of selective pressure, is distributed in genetic variants involved in height. iHS is a score specifically oriented to detect recent adaptive phenomena with higher geographical resolution. We found that, in each population, the number of SNPs associated with height having a value of iHS falling in the highest 5% of the genomic distribution is compatible with the expectation (varying from 4-7% across populations; Figure 2). This finding seems to indicate that, even if recent local adaptation phenomena cannot be excluded, their role seems to be marginal.

Another hypothesis that we explored was that loci responsible of local adaptive phenomena on different phenotypes, in linkage disequilibrium with height related variants, were responsible of the selective signatures that we found. Under this hypothesis, the increased mean FST value that we attributed to the height associated genes could be the by-product of selective pressures acting on other traits. To test this hypothesis, we extracted height related SNPs that were in linkage disequilibrium (r^2^>0.8 in at least one population) with alleles associated to other traits in genome-wide studies. We found 11 SNPs, 9 of which belonging to the “core set” (Table S1). As far as we know, there are no clearly identified signals of selective pressure on the phenotypes in linkage disequilibrium with the height associated SNPs. Moreover, also removing these SNPs from the analysis, the “core set” still show a significant higher F_ST_ (median = 0.099 vs. 0.086; p = 0.03452, one-tailed Mann-Whitney test).

We are aware that the increase in the mean F_ST_ value could be, at least in part, due to an eventual difference across the mean heights of the three populations considered. In other words, the increase in the mean F_ST_ value can be divided in two distinct components, where the first one accounts for the differences in the mean height while the second one accounts for the pure genetic differences. Unfortunately, at the best of our knowledge, data regarding the mean height of each population are not present in literature so far. For this reason, we were unable to estimate the relative weight of the two components or exclude the effect of the first one. Moreover, it is worth stressing that the 180 associated SNPs found by Lango Allen and colleagues only explain about 10% of the variance in adult height [8]. For this reason, wherever present, phenotypic differences among populations should have a marginal effect.

In conclusion, with the increasing availability of genomic data, the study of adaption of polygenic traits is becoming possible at experimental level. The lack of methods and models specifically tailored to this kind of study still leave it a great challenge. In this work we found that in the particular case of the height signals of population differentiation are present but, conversely from “hard sweeps”, they are small and distributed as recently hypothesized. Moreover, we suggest that, for this specific trait, mechanisms of spontaneous symmetry breaking seem to be a reasonably model to explain, at least in part, its evolutionary dynamic.

## Materials and Methods

### Data and statistical analysis

Analysis is based on the HapMap Public Release #27 (merged II+III) datafiles. We analyzed data from the CEPH (Utah residents with ancestry from northern and western Europe), Yoruba in Ibadan, Nigeria (YRI), Han Chinese in Beijing, China (CHB) and Japanese in Tokyo, Japan (JPT) samples. We pooled the CHB and JPT samples to form a single sample. Additional SNP information about physical positions and SNP-gene association were obtained from dbSNP build 129. We excluded by this analysis SNPs that were either non sampled or non polymorphic in all the three samples. We also excluded SNPs with a minor allele frequency <5% in all of the 3 samples. Per-population linkage disequilibrium data (r^2^) were obtained from HapMap Public Release #27 (merged II+III) as well. F_ST_ was calculated using the unbiased estimator proposed by Weir and Cockerham as in Amato et al [15]. All data was merged in a local MySQL database. As “genomic background” we refer to all the SNPs in this database, for a total of 3,294,557 SNPs.

Variants associated with height were collected from [8]. Of the 180 provided SNPs, 176 were present in our database and hence considered in our analysis.

The normalized iHS scores were obtained from UCSC Genome Browser “HGDP iHS” track. They were calculated using SNPs genotyped in 1,043 individual coming from 53 populations worldwide by the Human Genome Diversity Project in collaboration with the Centre d'Etude du Polymorphisme Humain (HGDP-CEPH). The 53 populations were divided into seven continental groups: Africa (Bantu populations only), Middle East, Europe, South Asia, East Asia, Oceania and the Americas. Per-SNP iHS scores were smoothed in windows of 31 SNPs, centered on each SNP.

Data on the association of SNPs with diseases was obtained from a catalog of genome wide association studies available at http://www.genome.gov/gwastudies, (accessed 12/13/10).

All statistical analyses were performed with R ver. 2.10 (R Foundation for Statistical Computing, Vienna, Austria; http://www.r-project.org/) considering 0.05 as significance threshold.

### Simulations

We simulated three populations of diploid organisms of fixed sample size evolving independently each other. Individuals are represented by 20 markers, where half of them are assumed to be neutral, and the remaining ones contribute additively and uniformly to the phenotype in a codominant way. Basically, markers evolve under a Wright-Fisher model with recombination. Polygenic selection is then simulated through viability selection. Denoting with *m* the number of beneficial alleles carried by an individual, each contributing with *x* to the phenotype, the fitness is parameterized as *exp*(-(*m*-µ)^2^ *x* ^2^/(2*s*) ). In the previous expression the quantity *s* measures the selection strength and µ is the number of beneficial alleles that maximizes the fitness. In figure S1 it is shown a particular case where the sample size is N = 10,000, *s* = 10, µ = 10 and *x* = 5. Furthermore, the initial allele frequency is set to 0.5 for all markers. Lower values of N increase the effect of genetic drift, while different values of µ change the number of alleles rising in frequency in each population. By tuning the value of *x* ^2^/*s* one can change the strength of selection hence affecting the time required to observe relevant variation in allele frequencies. Nevertheless, these changes do not qualitatively affect the results.

## Supporting Information

Figure S1 **Trajectories of the allelic frequencies for markers under polygenic selection in three simulated populations.** Each column, i.e. top and bottom panel together, represents a different population. Top panels show trajectories over time for the set of 10 alleles under polygenic selection; bottom panels show trajectories for set of 10 neutral alleles. Different colors mark different alleles, consistently across populations.
(TIFF)

Table S1 **Variants associated to height in linkage disequilibrium (r^2^>0.8 in at least one population) with alleles associated with other traits in genome-wide association studies.** For each height variant is reported the GWAS variant in linkage, the trait and the PubMed ID for the study itself.
(XLS)

## References

[1] Nielsen R, Hellmann I, Hubisz M, Bustamante C, Clark AG (2007). Recent and ongoing selection in the human genome. Nature reviews.. Genetics.

[2] Sabeti PC (2006). Positive Natural Selection in the Human Lineage.. Science.

[3] Stearns SC, Byars SG, Govindaraju DR, Ewbank D (2010). Measuring selection in contemporary human populations. Nature reviews.. Genetics.

[4] Holsinger KE, Weir BS (2009). Genetics in geographically structured populations: defining, estimating and interpreting F(ST). Nature reviews.. Genetics.

[5] Roff DA (1997). Evolutionary Quantitative Genetics..

[6] Hancock AM, Alkorta-Aranburu G, Witonsky DB, Di Rienzo A (2010). Adaptations to new environments in humans: the role of subtle allele frequency shifts. Philosophical transactions of the Royal Society of London.. Series B, Biological sciences.

[7] Pritchard JK, Pickrell JK, Coop G (2010). The genetics of human adaptation: hard sweeps, soft sweeps, and polygenic adaptation.. Current biology: CB.

[8] Lango Allen H, Estrada K, Lettre G, Berndt SI, Weedon MN (2010). Hundreds of variants clustered in genomic loci and biological pathways affect human height.. Nature.

[9] Nettle D (2002). Women's height, reproductive success and the evolution of sexual dimorphism in modern humans. Proceedings.. Biological sciences/The Royal Society.

[10] Nettle D (2002). Height and reproductive success in a cohort of british men.. Human Nature.

[11] Pawlowski B, Dunbar RI, Lipowicz A (2000). Tall men have more reproductive success.. Nature.

[12] Pritchard JK, Di Rienzo A (2010). Adaptation – not by sweeps alone.. Nature Reviews Genetics.

[13] Sayama H, Kaufman L, Bar-Yam Y (2000). Symmetry breaking and coarsening in spatially distributed evolutionary processes including sexual reproduction and disruptive selection. Physical review.. E, Statistical physics, plasmas, fluids, and related interdisciplinary topics.

[14] Doorn GS van, Dieckmann U (2006). The long-term evolution of multilocus traits under frequency-dependent disruptive selection.. Evolution; international journal of organic evolution.

[15] Amato R, Pinelli M, Monticelli A, Marino D, Miele G (2009). Genome-wide scan for signatures of human population differentiation and their relationship with natural selection, functional pathways and diseases.. PloS one.

