# Supplementary figures and images for: Signs of Selective Pressure on Genetic Variants Affecting Human Height
Source: PLoS One. 2011 Nov 9;6(11):e27588. doi: 10.1371/journal.pone.0027588 (PMC3212575; doi:10.1371/journal.pone.0027588)

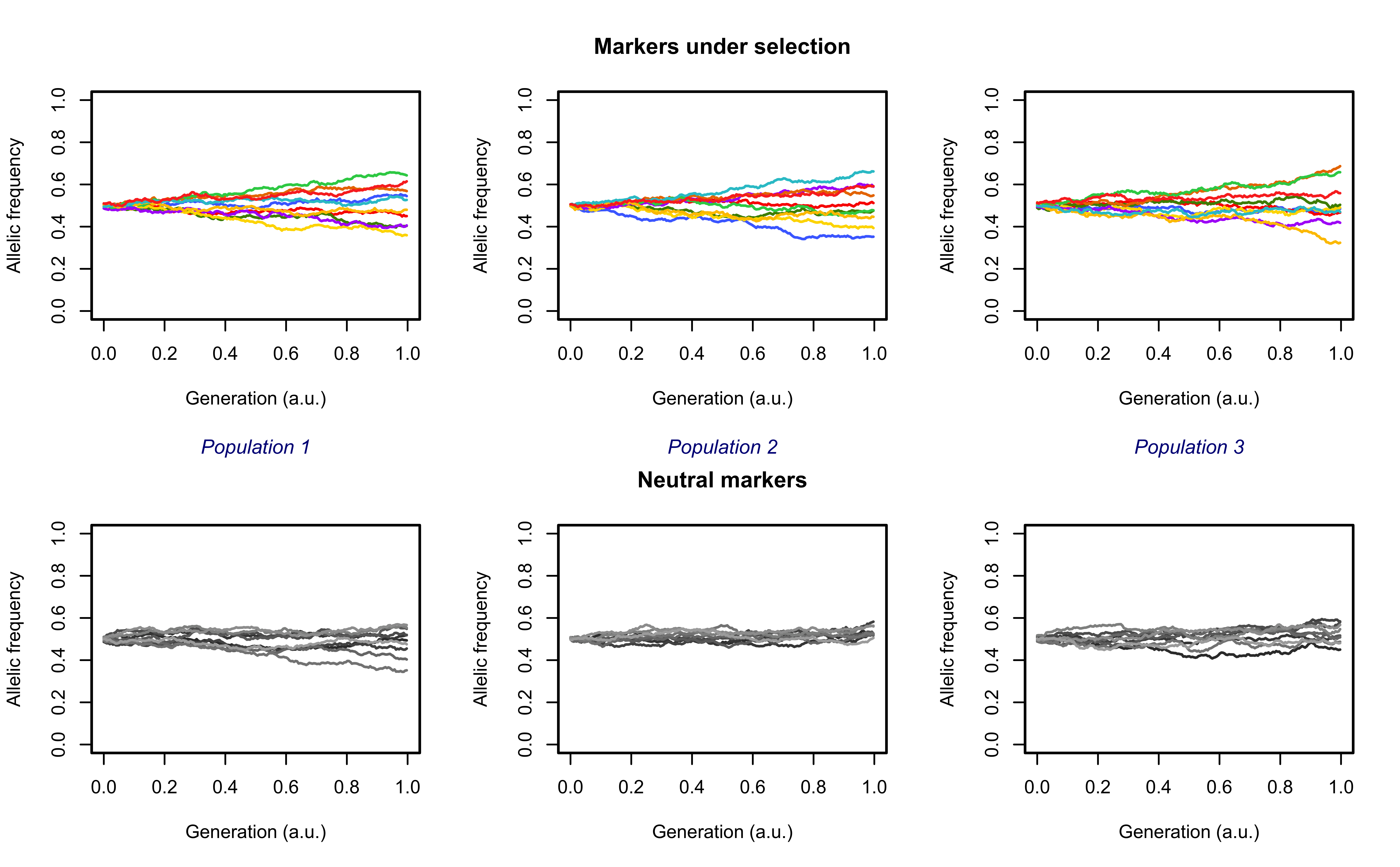

Supplement: Figure S1 — Trajectories of the allelic frequencies for markers under polygenic selection in three simulated populations. Each column, i.e. top and bottom panel together, represents a different population. Top panels show trajectories over time for the set of 10 alleles under polygenic selection; bottom panels show trajectories for set of 10 neutral alleles. Different colors mark different alleles, consistently across populations. (TIFF) [file pone.0027588.s001.tiff]
